# Using virtual reality to estimate aesthetic values of coral reefs

**DOI:** 10.1098/rsos.172226

**Published:** 2018-04-18

**Authors:** Julie Vercelloni, Sam Clifford, M. Julian Caley, Alan R. Pearse, Ross Brown, Allan James, Bryce Christensen, Tomasz Bednarz, Ken Anthony, Manuel González-Rivero, Kerrie Mengersen, Erin E. Peterson

**Affiliations:** 1 ARC Centre of Excellence in Mathematical and Statistical Frontiers, Queensland University of Technology, Brisbane, Australia; 2 School of Electrical Engineering and Computer Science, Queensland University of Technology, Brisbane, Australia; 3 Visualisation and eResearch, Institute for Future Environments, Queensland University of Technology, Brisbane, Australia; 4 Institute for Future Environments, Queensland University of Technology, Brisbane, Australia; 5 Global Change Institute, The University of Queensland, Brisbane, Australia; 6 ARC Centre of Excellence for Coral Reef Studies, The University of Queensland, School of Biological Sciences, St Lucia, Queensland, Australia; 7 Australian Institute of Marine Science, Townsville, Australia

**Keywords:** landscape changes, monitoring, remote ecosystems, beauty, human senses, Bayesian modelling

## Abstract

Aesthetic value, or beauty, is important to the relationship between humans and natural environments and is, therefore, a fundamental socio-economic attribute of conservation alongside other ecosystem services. However, beauty is difficult to quantify and is not estimated well using traditional approaches to monitoring coral-reef aesthetics. To improve the estimation of ecosystem aesthetic values, we developed and implemented a novel framework used to quantify features of coral-reef aesthetics based on people's perceptions of beauty. Three observer groups with different experience to reef environments (Marine Scientist, Experienced Diver and Citizen) were virtually immersed in Australian's Great Barrier Reef (GBR) using 360° images. Perceptions of beauty and observations were used to assess the importance of eight potential attributes of reef-aesthetic value. Among these, heterogeneity, defined by structural complexity and colour diversity, was positively associated with coral-reef-aesthetic values. There were no group-level differences in the way the observer groups perceived reef aesthetics suggesting that past experiences with coral reefs do not necessarily influence the perception of beauty by the observer. The framework developed here provides a generic tool to help identify indicators of aesthetic value applicable to a wide variety of natural systems. The ability to estimate aesthetic values robustly adds an important dimension to the holistic conservation of the GBR, coral reefs worldwide and other natural ecosystems.

## 1. Introduction

Coral reefs are the most species-rich ecosystems in the ocean [1], providing food for people [2], habitat for hundreds of thousands of marine species [3], coastline protection from wave exposure [4], and recreational and cultural heritage benefits [5]. In addition, their exceptional natural beauty generates cultural ecosystem services estimated at USD 110 000 ha^−1^ annually [6]. This estimate is greater than any other ecosystem on the Earth and highlights the importance of coral reefs in terms of ecosystems services and associated benefits they provide to human society [7]. However, their recent physical degradation globally may also have reduced their aesthetic value. The loss of aesthetic ecosystem services could compromise reef conservation efforts [8–10] if it causes people to disengage from reef conservation [11].

Australia's Great Barrier Reef (GBR) was designated a World Heritage Area in 1981 and is widely recognized for ‘containing superlative natural phenomena or areas of exceptional natural beauty and aesthetic importance’ (Criterion vii) [12]. Under the World Heritage Convention, the Australian Federal and Queensland State governments are obliged to monitor and report on the GBR's aesthetic services, as well as more traditional ecosystem-health measures such as water quality and biodiversity. A large number of visual criteria have been evaluated as part of a GBR aesthetic-value assessment in an effort to identify attributes that embody the values described in Criterion vii [13]. However, these methods do not capture other non-visual, experiential aspects of beauty [13–15].

The perception of beauty is much broader than visual satisfaction; it also includes the sense of pleasure evoked by experiences related to sight, touch, sound, taste or smell [10,13]. For example, the way that the GBR's aesthetic value is described has changed through time as new technologies have become available [15]. When reef tourism began in the 1920s, beauty was described using human senses such as the smell of trees, the sound of the wind and even the taste of the water. As scuba-diving and cameras became popular, visual reef aesthetics above and below the water were more commonly reported. More recently, high-definition video and imagery have become widely available, allowing people to remotely view the reef. Despite the widespread availability of these visual resources, it is still difficult to identify what exactly makes the GBR aesthetically pleasing, thereby precluding quantitative evaluation of aesthetic attributes. Thus, new methods are needed that capture both visual and experiential aspects of reef aesthetics [11,16].

Immersion via virtual reality (VR) is the process of replacing real sensory input with inputs from a computer system, such that the person is unaware of their outside reality. Immersive VR environments can be 360° images or synthetic three-dimensional environments, using head mounted displays responding to user movements. Immersion in VR places a person in a situation similar to where their knowledge was developed, thereby activating emotions and knowledge linked to those past experiences [17]. As a result, VR can produce better qualitative information compared with traditional surveys of expert knowledge [17,18]. For example, VR applications have recently been developed to elicit information from experts about habitat suitability for the rock wallaby (*Petrogale penicillata*) in Australia [17] and jaguars (*Panthera onca*) in Peru [19]. However, to our knowledge, VR has never been used to elicit information about ecosystem-level aesthetic attributes.

Accordingly, we developed a VR platform using 360° imagery from the GBR and used it to elicit information about people's perceptions of reef beauty. We interviewed three groups of observers with different experiences of coral reefs: (i) Marine Scientist, composed of experts in coral-reef ecology with extensive personal experience and scientific knowledge of coral reefs; (ii) Experienced Diver, who have extensive diving experience in coral-reef environments; and (iii) Citizen, who for the most part have only experienced coral reefs through documentaries and images. The goals of the elicitation were to (i) identify quantifiable attributes of reef aesthetics and (ii) determine whether these attributes differed among the groups of observers elicited. A Bayesian hierarchical logistic regression model [20] was developed to quantify the relationship between aesthetic attributes and the overall perception of an aesthetically pleasing reef, which also provided insights about the relationship between past experiences and observers' sense of beauty.

## 2. Methods

### 2.1. Image selection and clustering

**Figure 1.**
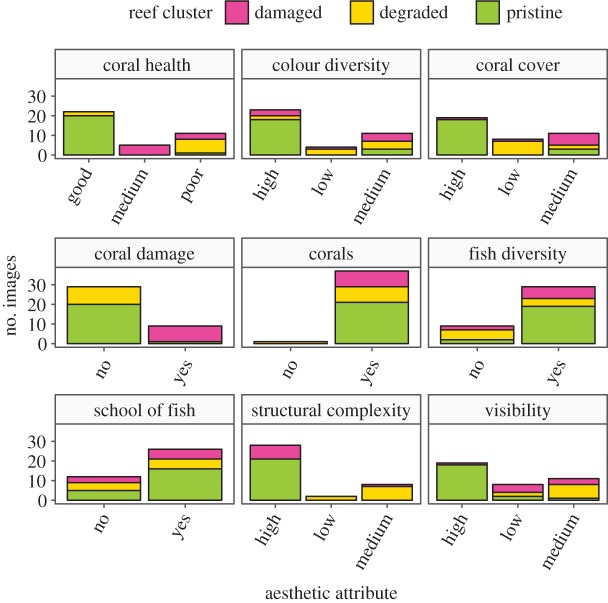
Characteristics of aesthetic attributes for each reef cluster. Clusters were determined using hierarchical clustering and aesthetic attributes from the literature.

A total of 39 360° images collected throughout the GBR in 2012 were provided by the XL Catlin Seaview Survey [21]. The images represented a range of attributes that are believed to describe the aesthetic value of the GBR [13] and defined as part of a previous assessment of the GBR World Heritage Area in 2013. These attributes included coral cover and structural complexity, coral health, colour range, damage to corals, fish abundance and diversity, as well as visibility (i.e. water clarity). The 39 images were categorized into three clusters (i.e. reefs in pristine, damaged and degraded states) using hierarchical clustering and these reef-aesthetic attributes (figure 1). The pristine reef cluster was characterized by high colour diversity, abundant fish, high coral cover without apparent damage and high levels of structural complexity and visibility. Images from the damaged reefs cluster were characterized by poor to medium coral health, and moderate coral cover, but high habitat complexity. The degraded reef cluster contained images with low to medium reef structural complexity, poor to medium visibility and low coral cover with generally poor coral health.

We then developed a random sampling design, stratified across each of the reef clusters [22] that ensured that (i) each observer's perceptions and observations were elicited during viewing of at least one image from each of the three clusters, (ii) the order of the images presented to an observer were varied among the participants, and (iii) each image was used for elicitation approximately the same number of times (see electronic supplementary material, ESM1). The package cluster [23] was used for hierarchical clustering and all analyses were undertaken in the R statistical software [24].

### 2.2. Elicitation of reef aesthetics

Based on the reef-aesthetic attributes described above, we prepared eight questions with yes/no responses using non-technical language that could be easily understood by all observer groups (table 1). Note that some reef aesthetics attributes such as coral cover and coral health were not used in the interview because they were considered too technical to be properly understood across all observer groups. These attributes were replaced with questions about the presence of individual fish and biodiversity other than corals and fish. For each question, the participants were also asked about the uncertainty associated with their answers (i.e. sure, medium sure and unsure). In addition, demographic information about the participants was collected (table 1).

**Table 1.** Information elicited from each participant during the interview.

variable	description
*aesthetics interview*	
Q1. Beauty	do you find the image visually pleasant?
Q2. Visibility	water quality: is the image hazy?
Q3. Structural complexity	structural complexity: do the live corals on the reef form structurally complex habitats?
Q4. Coral damage	damage on the reef: can you see evidence of damage to the reef?
Q5. Colour diversity	is the reef mostly one colour?
Q6. Individual fish	do you see individual fish?
Q7. School of fish	do you see schools of fish?
Q8. Fish diversity	do you see more than one type of fish?
Q9. Biodiversity	can you see organisms other than corals or fish?
*demographics*	
group	participant belongs to the Marine Scientist, Experienced Diver or Citizen group
gender	gender of the participant, male or female
age	participant belongs to the class 16–25, 26–45 or over 45 years of age
dive experience	participant that never dived, used to dive occasionally (less or equal to one time per year) or often (more than one time a year)

### 2.3. Elicitation using virtual reality

We used VR to elicit information about reef aesthetics from a total of 37 Marine Scientists, 32 Experienced Divers and 36 Citizens between late September and mid-November 2016. The 105 participants represented a range of ages (18 to over 45), gender (61% male and 39% female) and underwater experiences (32 participants had never scuba-dived, 34 dived occasionally and 39 frequently scuba-dived). Each of the participants was given a training document to read, which described the interview questions (see the electronic supplementary material, ESM1). The elicitation sequence always started with the same ‘training image’, which was selected because its aesthetic attributes fell into medium-range categories. The reef habitat presented in the training image was characterized by high visibility, structural complexity and colourfulness. Its coral cover and health were low to medium (0–20% coral cover) [25], with no visible coral damage, schools of fish or a variety of fish species, but soft corals were present. Participants were subsequently shown four additional images of reefs, one pristine, two damaged and one degraded, presented in a randomized order. Participants answered each question from an immersive VR platform by selecting response choice buttons presented on the VR visual field, and their answers were uploaded to an online database. We also asked participants to verbally state their answers, which were recorded and cross checked with the database entries. The full elicitation of each participant took approximately 35 min to complete.

### 2.4. Reef aesthetics model

We constructed a hierarchical Bayesian logistic regression model to quantify aesthetic values of these images based on the relationships between aesthetic perception and attributes elicited from the participants (see the electronic supplementary material, ESM2). In total, eight aesthetic and five demographic attributes were used as explanatory variables (table 1). We then used the model to estimate aesthetic value expressed in terms of the probability of a reef being aesthetically pleasing as a function of the elicited data, observer groups (Marine Scientists, Experienced Divers and Citizens) and different levels of uncertainty (electronic supplementary material, appendix S2). Very few participants categorized their answers as ‘unsure’. Therefore, the unsure and medium-sure levels were pooled so that only two levels of uncertainty were included in the model (i.e. sure and less sure). Bayesian modelling was performed using the JAGS software called by the rjags package [26].

## 3. Results

### 3.1. Is this reef aesthetically pleasing?

**Figure 2.**
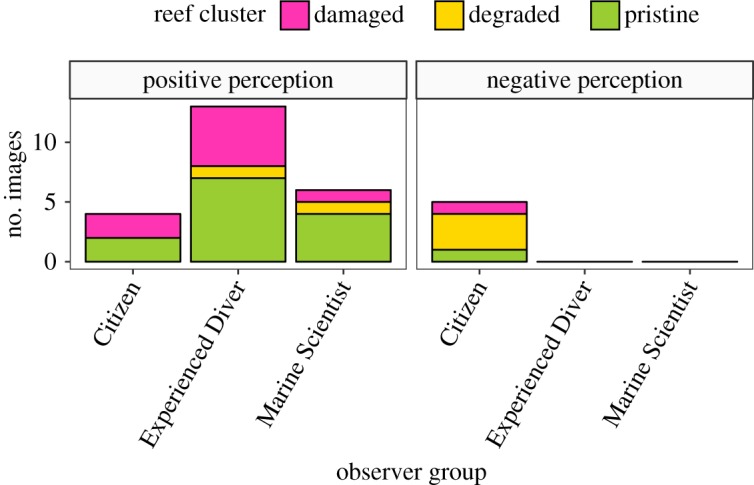
The number of images consistently classed as visually pleasant (i.e. positive perception) or visually unpleasant (i.e. negative perception), classed by observer group. A reef cluster was assigned to each image prior to the experiment.

Overall, the participants found most images aesthetically pleasing (table 2). At the group level, the Experienced Divers were most likely to answer ‘yes’, while the Citizens had the highest proportion of ‘no’ responses. Experienced Divers were never ‘unsure’ about their answers, whereas the Marine Scientists were unsure the most often (table 2). In total, 60% of participants found the training image visually pleasant, and 65% indicated that they were sure about their answer. Only one participant stated that the reef in the training image was aesthetically unpleasant, but that person was also unsure about his response. Ten images were unanimously deemed visually pleasing across all groups, while only one image was unpleasant to all participants. Not surprisingly, all images deemed pleasing were of reefs within the pristine cluster, and the image unanimously deemed unpleasant was from the degraded cluster. We also observed patterns within groups about aesthetically pleasant or unpleasant reefs. The Experienced Divers were the most unanimously positive with 13 reefs being described as aesthetically pleasant followed by the Marine Scientists with six reefs. However, the Citizens were the only group to unanimously agree that some of virtual reefs were aesthetically unpleasant (figure 2) and most of these five reefs belonged to the degraded cluster.

**Table 2.** The proportion of responses by Marine Scientists, Experienced Divers and Citizens to the question, ‘do you find this place aesthetically pleasing?’ and related to uncertainty of their answers.

observer group	responses	proportion (%)
*do you find this place aesthetically pleasing?*		
Marine Scientists	yes	34.1
	no	34.6
Experienced Divers	yes	33.2
	no	25.7
Citizens	yes	32.6
	no	39.6
*how sure are you?*		
Marine Scientists	unsure	2.8
	medium sure	24
	sure	73.2
Experienced Divers	unsure	—
	medium sure	23.1
	sure	76.9
Citizens	unsure	1.1
	medium sure	33.3
	sure	65.6

### 3.2. Attributes of aesthetically pleasant reefs

**Figure 3.**
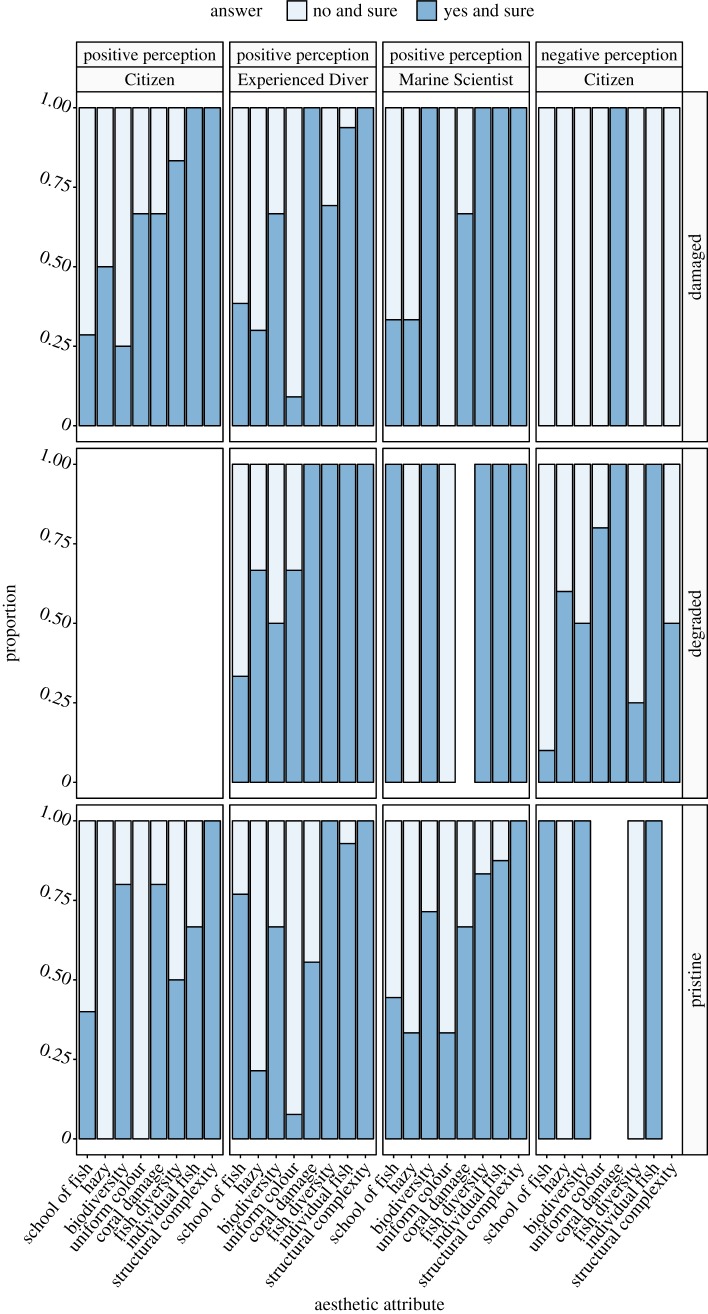
The proportion of answers about reef-aesthetic attributes for the images unanimously classified as visually pleasant (i.e. positive perception) or visually unpleasant (i.e. negative perception) by observer group (Citizen, Experienced Diver and Marine Scientist), for each reef cluster (damaged, degraded and pristine). Only the responses ‘no and sure’ and ‘yes and sure’ are displayed. In places where data are missing, the participants were either unsure or medium sure about their responses.

For the reef environments that were unanimously perceived as pleasant or unpleasant, we examined how the three groups of participants scored aesthetic attributes between the three reef clusters (figure 3). In this case, we focused on the answers that were ‘yes and sure’ and ‘no and sure’, noting that they made up 76% of responses. For the reefs that were deemed pleasant (i.e. positive perception), all participants observed individual fish, regardless of the reef clusters (table 1, Q6). However, this was not surprising because the presence of fish was not used in the sampling design and as such, they were present in all images. High structural complexity (table 1, Q3) was one of the main criteria associated with a positive perception irrespective of the reef clusters. When the participants from the Marine Scientists group of observers answered that a reef was aesthetically pleasing, it was unanimously deemed structurally complex. For the unpleasant reef (i.e. negative perception), the Citizens answered in the same way to describe the reefs from the damaged and pristine reef clusters. The damaged reef was characterized by an absence of fish and other organisms, as well as the presence of coral damage. By contrast, the most notable pattern in responses for the pristine reef classified as unpleasant by the Citizens was the lack of certainty in the participants' responses related to structural complexity, coral damage and diversity of colours (figure 3, missing bars).

### 3.3. Reef-aesthetic model

**Figure 4.**
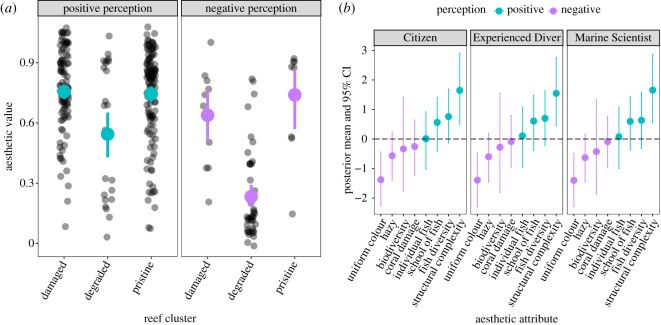
(*a*) Estimated coral-reef-aesthetic values for images unanimously deemed aesthetically pleasing (i.e. positive perception) and those deemed aesthetically unpleasant (i.e. negative perception) by reef cluster. Note that the points are jittered on both axes. (*b*) Model parameter estimates describing the relationship between the aesthetic attributes and aesthetic value by observer group. Dots represent the posterior mean and lines the 95% CI around those means. Aesthetic attributes represented by blue dots show a positive influence on aesthetic value and attributes in purple dots a negative influence. Positive and negative perceptions are deduced using the sign of the posterior mean of the parameters.

The purpose of the model was to estimate the probability that an observer found a reef aesthetically pleasing (i.e. the aesthetic value of a coral reef was high) and to quantify the relationship between this response and the explanatory variables representing aesthetic attributes and demographic variables (table 2). The highest aesthetic value was 0.95 for a 360° image from the pristine cluster, while the lowest was 0.18 for an image from the degraded reef cluster. We also examined aesthetic values estimated by the model by reef cluster for images that were unanimously perceived as pleasant (i.e. positive perception) and unpleasant (i.e. negative perception) by observers (figure 4). In both cases, aesthetic values from the degraded reef cluster were the lowest, with an average of 0.54 and 0.23 for the positively and negatively perceived images, respectively. Surprisingly, we did not find a strong difference between estimated aesthetic values for images within the damaged and pristine reef clusters. Note that the main difference between the pristine and damaged clusters relates to coral damage with similar structural complexity (figure 1). Two aesthetic attributes had 95% CIs that did not include zero, suggesting that they describe attributes strongly related to reef-aesthetic value. Structural complexity (table 1, Q3) was positively associated with aesthetic value, while the lack of colour diversity was negatively associated with aesthetic value (table 1, Q5; figure 4). The 95% CIs for hazy (i.e. low visibility), coral damage, fish presence and biodiversity (table 1, Q2, Q4, Q6–Q8 and Q9, respectively) included zero (figure 4), indicating that these are not significantly associated with perception of aesthetic value. Most of the parameter estimates for the demographic explanatory variables were similar across the three groups of interviewees. Participants younger than 26 years old, occasional divers and participants from the oldest age class tended to be more positive in their responses but the 95% CIs for these effects also included zero. No effect of gender was detected at 95% CI.

## 4. Discussion

Aesthetic ecosystem services describe the human psychological well-being and satisfaction provided by beautiful ecosystems [8,27]. However, this dependence of humans on natural environments is threatened by the global degradation of ecosystems to the point that the benefits to humans may also be impaired [9,28,29]. Ideally then, we would like to monitor the status and trends in aesthetic value along with other more traditional ecosystem-health indicators. However, our ability to identify and quantify exactly what makes an ecosystem aesthetically pleasing is insufficient at present. The methods presented here begin to address this important knowledge gap.

Our results showed that high structural complexity of coral reefs strongly increased their aesthetic value. Structurally complex reefs are also considered healthy because they provide a diverse array of habitats. In addition, the loss of structural complexity has detrimental effects on biodiversity [30–33]. Marine Scientists are largely aware of this relationship and, therefore, their perception of beauty may have been linked to what they perceive as a healthy reef. Their consistent answers and language during the interviews also suggested this was true. Although Citizens and Experienced Divers would likely be less aware of this relationship, they also found structurally complex reefs pleasing. Thus, structural complexity may be a good indicator of aesthetic value *and* reef health irrespective of who is observing a coral reef.

Coral-colour diversity was also positively associated with high reef-aesthetic value, which confirms assumptions made in previous reef-aesthetic studies that humans prefer colourful reefs [11,34,35]. However, the relationship between coral-colour diversity and coral health is not straightforward. The Coral Watch citizen-science programme measures coral colour using a Coral Health Chart to assess the degree of bleaching in coral colonies [36]. However, a colourful reef is not always healthy. For example, fluorescing coral in the initial stages of bleaching display intense colours [37], while other colourful reef organisms such as sponges and soft corals are often prevalent in stressed coral ecosystems [38,39]. Although colour diversity and intensity can inform management and conservation with respect to aesthetic services [28], the lack of a broad-based relationship between reef colour and health means that it must be assessed within the context of other reef-aesthetic attributes and health indicators, while keeping this limitation in mind.

We found no evidence supporting our initial hypothesis that a person's past experiences influence their perception of beauty. Although this may be due to the relatively small number of observers (maximum of 37 per group), there was a significant amount of agreement across groups about attributes of reef aesthetics. Additionally, our results were similar to those of Dinsdale & Fenton [11], who found that current human perception of aesthetic value may be blurred by the social beliefs of an ‘ideal’ coral-reef environment. In our study, Citizens were the most sensitive to images of degraded coral reefs, which were characterized by poor visibility and low coral health. While technologies such as high-definition underwater cameras and lighting equipment have modified reef-aesthetic values in the past [15], they may now have affected public expectation to a point where a healthy reef observed under natural light conditions is not considered aesthetically pleasing to the general public. Our observers may have also been conditioned to expect damaged reefs. Citizens identified coral bleaching on 33% of the images, compared to Experienced Divers and Marine Scientists, who identified bleached coral in 23% and 18% of the images, respectively (see the electronic supplementary material, ESM4). The extent and severity of the 2016 GBR coral-bleaching event was widely reported in the Australian media and the Citizens' familiarity with this issue was apparent from their comments. However, the images used in this study were taken in 2012 and none contained evidence of extensive coral bleaching. This suggests that participants' perception may have been negatively affected by media coverage, and that the Citizens were perhaps more strongly affected due to a lack of reef experiences with which to compare these images. We noticed this same effect with Marine Scientists, but to a lesser degree. Instead, their ecological knowledge seems to have influenced their responses, as the diversity of fish species and other organisms was one of the main characteristics they found unanimously pleasing. Thus, social expectations appear to influence people's perception of beauty, as these responses cannot be explained by their past experiences of coral reefs.

The combined use of VR and modern statistical modelling can be easily applied to elicit information in other domains for conservation purposes including social benefits and educational and environmental outreach opportunities for coral reefs, in addition to other similar remote ecosystems that are difficult or expensive to physically access. For example, the ability to immerse citizens into virtual coral reefs is likely to create new insights regarding coral-reef knowledge compared with two-dimensional images or high-resolution underwater video, which are typically shown in the media. In addition, the VR experience could be used to increase the public's understanding of environmental pressures on coral reefs [37] and their willingness to pay for the ecosystem services that reefs provide. Conservation strategies that include people's willingness to pay for environmental services have been implemented to encourage reforestation in Europe and South America [38]. Our findings also illustrate different uses of uncertainty levels among observer groups. Further investigations into the sources of these uncertainty levels and their impacts on cognitive systems will be useful for understanding the sense of beauty perceived by human observers. It will also show the benefits of using the VR experience compared with other more traditional elicitation methods such as online surveys and interviews on paper.

The framework developed here provides exciting new opportunities to study aesthetics as an ecosystem service [8,10,29]. Although natural beauty is, and will remain, somewhat intangible, we were able to capture people's perception of natural beauty and quantify attributes of coral-reef aesthetics. Colour diversity and structural complexity were the two important attributes associated with reef aesthetics. Note that structural complexity is already used as a reef-health indicator, while colour diversity is not. Thus, our results show that beauty and health are not one and the same in a coral-reef ecosystem. The promotion of functional beauty [40] based on knowledge of reef ecology and aesthetic services would add an important dimension to the holistic conservation efforts for the GBR and coral reefs worldwide, and also for the conservation of other natural ecosystems.

## Supplementary Material

Details on the reef aesthetics experiment

## Supplementary Material

Demographic information of the observers

## Supplementary Material

The 10 most frequent words for the question “Can you see evidence of damage to the reef?”

## Supplementary Material

Details on the Bayesian logistic regression model

## Data accessibility

Data are available through the Dryad Digital Repository at: https://doi.org/10.5061/dryad.94rt2 [41].
