# Supplementary material for: Using virtual reality to estimate aesthetic values of coral reefs
Source: R Soc Open Sci. 2018 Apr 18;5(4):172226. doi: 10.1098/rsos.172226 (PMC5936941; doi:10.1098/rsos.172226)
Supplement: Details on the reef aesthetics experiment [file rsos172226supp1.pdf]

ELECTRONIC SUPPLEMENTARY MATERIAL

Vercelloni J, Caley MJ, Clifford S, Pearse AR, Brown R, James A, Christensen B, Bednarz T, Anthony K, González-Rivero M, Mengersen K and Peterson E. **Using virtual reality to estimate aesthetic values of coral reefs**

1.1. The training document

Prior to elicitation, participants in the reef aesthetics experiment were provided with a training document. This document explained 1) the experiment, 2) the questions that participants would be asked and the criteria for answering them, and 3) the uncertainty grades attached to each response. Its purpose was to provide a summary of the experiment in a consistent manner for all participants.

Each participant was asked nine Yes-or-No questions within the VR interface, which were previewed in the training document. The definitions for concepts and question scope were designed to help the participant respond adequately to each question. In particular, the following were defined:

- Desirability

Desirability was defined as a personal appraisal by the participants regarding the beauty of the landscape that they were immersed in.

- Haziness

The concept of haziness was defined as being unable to see clear outlines of different objects in the distance.

- Structural complexity

Structural complexity was not rigidly defined; instead, participants were asked to view the reef from the perspective of a small fish. They were told that, if, as a small fish, they could find many places to hide in the reef, the reef should be considered a complex habitat. Participants were also asked to distinguish between topography and structural complexity; in other words, terrain with varied topographical features, such as hills, was not necessarily complex.

- Types of coral damage

Different types of coral damage (e.g. coral bleaching/disease, storm damage, fish predation, and pollution) were explained using images. Participants were also encouraged to describe other damage, even if those types of damage were not explained in the training document.

- Colours

The notion of colour intensity and diversity was addressed by asking about a lack of many colours within an image.

- Individual fish versus schools of fish

Individual fish were defined as fish that could easily be counted one-by-one, and fish were said to be schooling if there was a group of similar looking fish for which it would not be possible to quickly provide a count.

- Organisms other than corals or fish

For the study, corals were defined as hard corals and so questions relating to other organisms included soft corals. Additional examples of other organisms given to the participant were sea cucumbers, turtles, algae and sponges.

1.2. The training image

Each participant was shown five 360-degree images inside a Samsung GearVR headset, including one training image provided by the XL Catlin Seaview Survey (1). The training image was the same for all participants and was used to help standardise subsequent responses. Participants were asked to base their impression of all subsequent images on their judgements about the training image, which represented a medium-quality reef typical of the GBR.

1.3. Other images

After viewing the training image, the participants were shown four images from a pool of 38 images. The images were selected using a stratified random sampling design (2) in order to show observers reefs from each reef cluster. The sampling design was developed for up to 150 participants, m, 4 sample times, k_j , corresponding to the number of images shown during the interview (in addition to the training image) and 3 reef clusters, j.
To sample k_j times within cluster j for each of the m participants, a vector of the n_j image labels was permuted randomly, replicated $\lceil mk_j/n_j \rceil$ times and truncated to length mk_j. In this way, the maximum discrepancy between the numbers of times any pair of images would be allocated to the participants was 1. This vector was again permuted randomly and coerced to a matrix of dimension $m \times k_j$ and only kept as a valid design if no image was assigned to the same participant twice. This method was used to assign each participant one image from each of the degraded and damaged reef clusters, and two from the pristine reef cluster. The order of viewing these four images was randomly permuted for each user and all four were elicited after completing the elicitation of the training image. In this way, each image was viewed approximately the same number of times across the study design, with sequence effects removed via the permutation after ensuring every participant viewed the training image first.

1.4. Experiment progress

Six Samsung smart phones (the Samsung S5 and Samsung Note 6) were used for the experiment, using version 5 of the Android operating system. During the experiment, the phones were put on flight mode while connecting to a wireless internet connection and all apps other than the reef elicitation application were shut down to conserve battery power and to prevent overheating. Samsung GearVR headsets were used to perform the virtual reality (VR) experiments.

For each user to perform an elicitation for an image, a Quick Response (QR) code was generated to represent a unique media ID, which was a combination of user identifier (GUID) and image ID. The QR codes were generated and imported into R (R Core Team, 2016) for inclusion in a survey booklet that, for each participant, contained the five demographics questions, a full page print-out of each of the 5 QR codes, and two template pages for manual recording of the participant's answer to the nine elicitation questions and the sureness value. These were recorded and subsequently used to double

check the responses that were entered in the VR application and stored in the database. Using the GearVR headset, the participants scanned the QR code using phone camera, which initiated the elicitation session and triggered the download of the associated image over the phone internet connection. When they were finished with the elicitation for each image, participants were asked to submit their survey, which sent and stored the data in a database.

References

1. González-Rivero M, Beijbom O, Rodriguez-Ramirez A, Holtrop T, González-Marrero Y, Ganase A, et al. Scaling up Ecological Measurements of Coral Reefs Using Semi-Automated Field Image Collection and Analysis. Remote Sens. 2016 Jan;8(1):30.

2. Murray J V., Low Choy S, McAlpine CA, Possingham HP, Goldizen AW. The importance of ecological scale for wildlife conservation in naturally fragmented environments: A case study of the brush-tailed rock-wallaby (Petrogale penicillata). Biol Conserv. 2008;141(1):7–22.
