# Supplementary material for: Using virtual reality to estimate aesthetic values of coral reefs
Source: R Soc Open Sci. 2018 Apr 18;5(4):172226. doi: 10.1098/rsos.172226 (PMC5936941; doi:10.1098/rsos.172226)

ELECTRONIC SUPPLEMENTARY MATERIAL

Vercelloni J, Caley MJ, Clifford S, Pearse AR, Brown R, James A, Christensen B, Bednarz T, Anthony K, González-Rivero M, Mengersen K and Peterson E. Using virtual reality to estimate aesthetic values of coral reefs

Figure S1. Demographic information that was recorded for each observer before the experiment. Note that GBR stands for Great Barrier Reef.

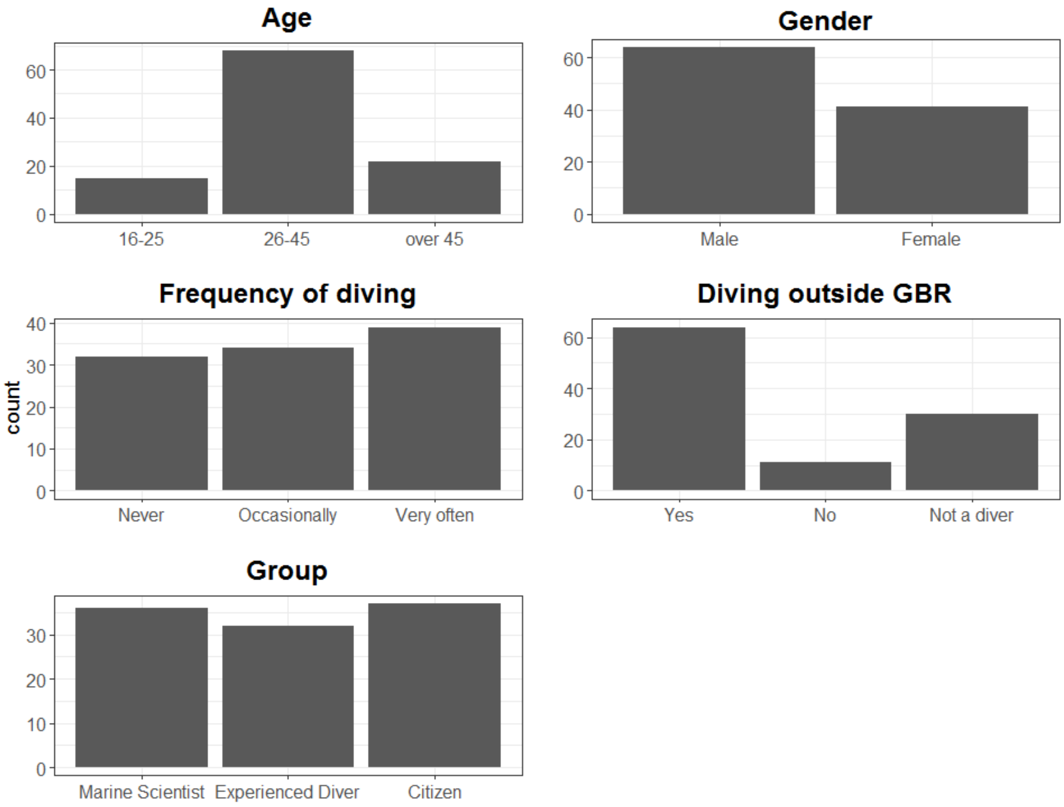

Supplement: Demographic information of the observers [file rsos172226supp2.pdf]
