# Supplementary material for: Using virtual reality to estimate aesthetic values of coral reefs
Source: R Soc Open Sci. 2018 Apr 18;5(4):172226. doi: 10.1098/rsos.172226 (PMC5936941; doi:10.1098/rsos.172226)
Supplement: The 10 most frequent words for the question “Can you see evidence of damage to the reef?” [file rsos172226supp3.pdf]

ELECTRONIC SUPPLEMENTARY MATERIAL

Vercelloni J, Caley MJ, Clifford S, Pearse AR, Brown R, James A, Christensen B, Bednarz T, Anthony K, González-Rivero M, Mengersen K and Peterson E. **Using virtual reality to estimate aesthetic values of coral reefs**

Figure S2. Wordclouds of the 10 most frequent words said by the three groups of observers for the question “Can you see evidence of damage to the reef?”. The size and colours relate to the frequency of each word.

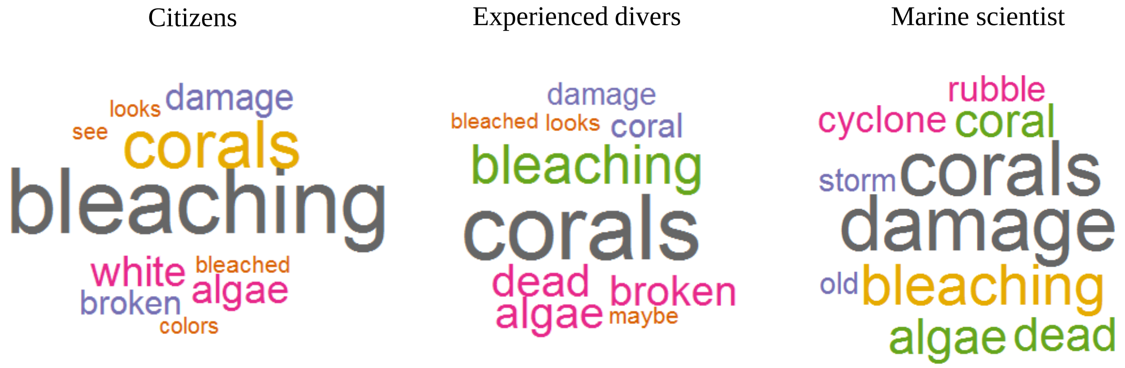

The R packages SnowballC (1) and wordcloud (2) were used for text stemming and generate the wordcloud while removing the punctuations, extra white spaces and English common stop words from the interviewers’ notes.

References

1. Bouchet-Valet M. SnowballC: Snowball stemmers based on the C libstemmer UTF-8 library [Internet]. 2014. Available from: https://cran.r-project.org/package=SnowballC

2. Fellows I. wordcloud: Word Clouds [Internet]. 2014. Available from: https://cran.r-project.org/package=wordcloud
