# Supplementary material for: Using virtual reality to estimate aesthetic values of coral reefs
Source: R Soc Open Sci. 2018 Apr 18;5(4):172226. doi: 10.1098/rsos.172226 (PMC5936941; doi:10.1098/rsos.172226)
Supplement: Details on the Bayesian logistic regression model [file rsos172226supp4.pdf]

ELECTRONIC SUPPLEMENTARY MATERIAL

Vercelloni J, Caley MJ, Clifford S, Pearse AR, Brown R, James A, Christensen B, Bednarz T, Anthony K, González-Rivero M, Mengersen K and Peterson E. Using virtual reality to estimate aesthetic values of coral reefs

A hierarchical logistic regression approach was used because of the nature of the response variable (i.e. binary data) and the complexity of the experiment (i.e multiple images and several reef clusters and groups of observers).

1.1. Model formulation

A hierarchical Bayesian logistic model was developed to estimate the probability that a reef is aesthetically pleasant as a function of multiple questions *k*, images *j*, groups *g* (Marine Scientists, Experienced Divers, and Citizens) and different levels of uncertainty *s* (medium and high).

In total, 5 demographic and 8 aesthetic attributes were used as explanatory variables. Here the baseline statement was the probability that a reef was deemed aesthetically pleasing if the responses were no to every question. Explanatory categorical variables have been centred at 0 and calibrated to use specific categories as the model baseline. In addition, the middle-age range [26-45] and the statement "I have never dived" were used as baselines to parameter estimation.

Data on reef aesthetics was modelled for each elicitation *i* based on the explanatory variables, x_{ik} and z_{ik} :

$$y_i \sim Bernoulli(p_i)$$

$$logit(p_i) = \alpha_j + \sum_{k=1}^{5} \psi_k z_{ik} + \sum_{k=1}^{8} \beta_{g,s,k}(x_{ik} - 0.5)$$

$$\beta_{g,s,k} \sim N(\beta_{g,k}, \tau_{s,k}), \forall g \in 1...3, \forall s \in 1...3, \forall k \in 1...8$$

$$\beta_{g,k} \sim N(\beta_k, \tau_{\beta_0})$$

$$\alpha_j \sim N(0, \tau_{\alpha_0}), \forall j \in 1...39$$

$$\psi_k, \beta_k \sim N(0, 10^{-2})$$

$$\tau_{s,k}, \tau_{\beta_0}, \tau_{\alpha_o} \sim \Gamma(10^{-2}, 10^{-2})$$

Reef-aesthetic attribute parameters, β, and demographic parameters, ψ, were considered differently. When the reef-aesthetic parameters were modelled for each question, the effects of group and uncertainty level were accounted for in the estimation. At the higher model level, reef-aesthetic parameters for each question, group and uncertainty were considered as random, with the mean estimated as function of groups, and variance components a function of uncertainty levels. The intercept parameters, α_j, was also random and estimated as a function of images with a variance term, τ_{α_0}. In contrast, the prior distributions for the demographic parameters were considered as independent normal distributions. The R package rjags (1) was used to fit the model.

1.2. Model diagnostics

The Markov Chain Monte Carlo (MCMC) chains included 90,000 iterations, with the first 5,000 discarded as the burn-in period, and 5,000 draws used to infer model parameters. MCMC convergence plots from the mcmcplots (2) and coda (3) packages were used to check posterior distributions of model parameters. Two approaches were used to estimate the model goodness-of-fit. First, we examined the distribution of the model residuals and did not find any evidence of outliers or lack-of-model fit (Figure S3). We also examined the relationship between the model residuals and the predictions and found that they were randomly distributed (Figure S4). Two main model formulations were compared; the model with the group effect (Citizens, Experienced Divers, and Marine Scientists) and a second model without a group effect. We compared the models and found that the model with the group effect had a lower Deviance Information Criterion (4) value than the model without the effect (445.9 and 463.4, respectively). This suggests that even though the parameter estimates are similar from group to group there are differences which further explain the variability in the reefs' aesthetic pleasance.

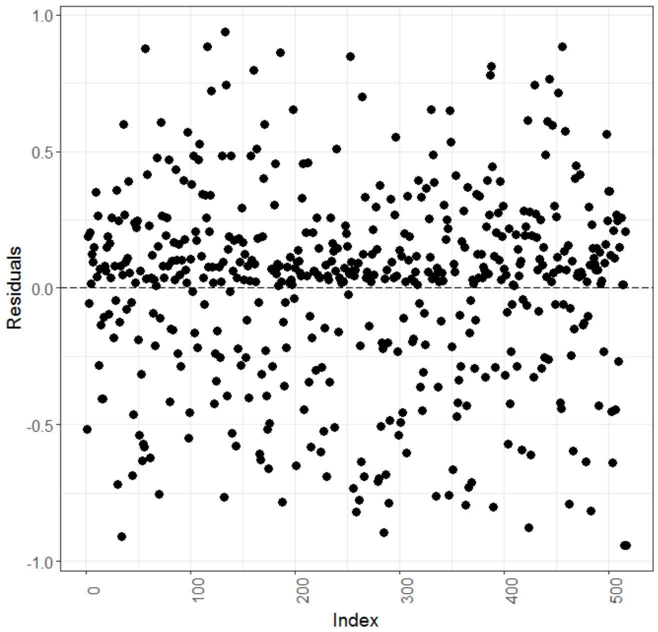

Figure S3. Distribution of the model residuals.

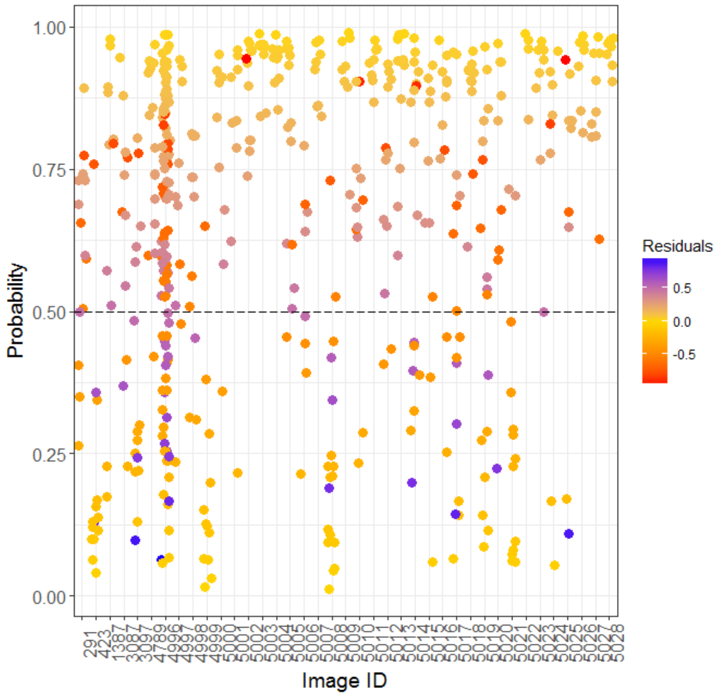

Figure S4. Estimated probabilities that an image is visually pleasant and their associated model residuals. Note that the points were jittered on the x-axis.

References

1. Plummer M. rjags: Bayesian Graphical Models using MCMC. R package version 4-6. https://CRAN.R-project.org/package=rjags. 2016.

2. McKay Curtis S. mcmcplots: Create Plots from MCMC Output [Internet]. 2015. Available from: https://cran.r-project.org/package=mcmcplots

3. Plummer M, Best N, Cowles K, Vines K. CODA: convergence diagnosis and output analysis for MCMC. R News [Internet]. 2006;6(March):7–11. Available from: http://cran.r-project.org/doc/Rnews/Rnews_2006-1.pdf#page=7

4. Spiegelhalter DJ, Best NG, Carlin BP, Van der Linde A. The deviance information criterion: 12 years on. J R Stat Soc Ser B Stat Methodol. 2014;76(3):485–93.
